# Pilot Testing a Photo-Based Food Diary in Nine- to Twelve-Year Old- Children from Dunedin, New Zealand

**DOI:** 10.3390/nu10020240

**Published:** 2018-02-20

**Authors:** Brittany K. Davison, Robin Quigg, Paula M. L. Skidmore

**Affiliations:** 1 Department of Human Nutrition, University of Otago, Dunedin 9054, New Zealand; brittany_davison@hotmail.com; 2 Cancer Society Social and Behavioural Research Unit, Department of Preventive and Social Medicine, Dunedin School of Medicine, University of Otago, Dunedin 9054, New Zealand; robin.quigg@otago.ac.nz

**Keywords:** children, dietary assessment, nutrients

## Abstract

The purpose of the study was to investigate if an Evernote app-based electronic food diary is an acceptable method to measure nutrient intake in children aged 9–12 years. A convenience sample of 16 nine- to twelve-year-olds from Dunedin, New Zealand, completed a paper-based food dairy on four days, followed by four more days using a photo-based diary on an iPod. This photo-based diary used a combination of photographs and short written descriptions of foods consumed. The photo-based diaries produced similar results to written diaries for all macronutrients and major micronutrients (e.g., calcium, fibre, vitamin C). Spearman correlation coefficients between the two methods for all nutrients, except sugars, were above 0.3. However, burden on researchers and participants was reduced for the photo-based diary, primarily due to the additional information obtained from photographs. Participating children needed less help from parents with completing the electronic diaries and preferred them to the paper version. This electronic diary is likely to be suitable, after additional formal validity testing, for use in measuring nutrient intake in children.

## 1. Introduction

A healthy diet is essential in childhood because it is associated with current and future health [1,2]. Therefore, dietary assessment methods appropriate for children are vital. Several traditional methods are used to measure energy and nutrient intake, including 24-h recalls, food frequency questionnaires (FFQs) and food records. The 24-h recall method is used in large-scale surveys in New Zealand, such as the New Zealand Adult Nutrition Survey [3]. An important limitation of this method is recall bias, where people cannot accurately remember everything they consumed [4]. Using 24-h recalls is especially difficult in children as several people may need to be interviewed to ensure all the food consumed is accurately reported [5]. For example, parents, teachers and friends’ parents may need to be consulted depending on where the child was on the specified day [5]. Children may not always be able to accurately describe what they have eaten. For similar reasons, while comprehensive self-completed FFQs are used commonly in large-scale studies of adults to determine long-term dietary intake [4,5], they are not ideal for comprehensive dietary assessment in children.

Weighed food diaries are the gold standard of dietary assessment, but are not suitable for children as this method is time consuming and places a high burden on participants and caregivers. Estimated food diaries are an alternative method, but accurate portion size estimation is an issue for children, requiring additional parental help [5]. A more recent method of collecting dietary data is using food photography. This involves participants taking photos of the food and drink they are going to consume, then another photo when they are finished [6]. A description of the photo can be added to provide extra information. The image can be used to determine what the person is eating and how much of it he/she ate. Benefits of this method are that portion size does not need to be estimated and there is a low participant burden [6]. Research suggests that younger people are more compliant with electronic nutrient data collection methods compared to paper-based methods (75% compared to 50% compliance) [7]. An increasing number of young people have smartphones, or other smart devices, therefore developing an electronic application (app) for these may be a cost-effective, low burden method of data collection. Previous U.S. research results suggest food photography is a valid and practical way to measure adult nutrient intake [8] and children’s food intake [9] when compared to food records. Therefore, the aim of this pilot study was to determine the acceptability of an Evernote app-based food diary (photo-based diary) on an iPod for measuring dietary intake in children, in comparison to traditional written food records, and to assess its usability in this population.

## 2. Materials and Methods

### 2.1. Subjects and Study Design

We aimed to recruit a convenience sample of 16 children aged nine to twelve years recruited from Dunedin, New Zealand via word of mouth. Investigators asked people they knew with children of eligible age or those who worked with children of eligible age to contact us for information if they wanted to take part. They in turn also spoke to parents of eligible children about the study.

Only children who (a) were literate and therefore able to complete the diaries, either on their own, or with help from parents, (b) were available throughout the study period (i.e., not going away from Dunedin during the study period) and (c) gave permission for audio-recording of the group interview (where applicable) were eligible to participate.

Parents and children were required to provide written informed consent before entering the study. Ethical approval for the study was obtained from the University of Otago Human Ethics Committee (Ref 13/265, 20 September 2013).

### 2.2. Data Collection

We used the sequential explanatory mixed method for this study. Participants and their caregiver met with a researcher and were first given a written food diary, as the reference method, to record all food and drinks consumed over 4 days. Child/parent pairs were given verbal instructions on how to complete the diary, and written instructions were contained within the diary. Both of these were tailored to be understood by children of eligible age. Child participants were asked to complete the diary on 4 non-consecutive days, including a weekend day, with help from their primary caregiver if necessary. Participants were asked to include brand names of food and drinks to improve the accuracy of the final results. Where, possible the researcher would meet with the participant and their parent after the first day of recording to ensure all the necessary information was recorded.

A few days after completing the written food diary, each participant was given an iPod with the Evernote app on it. The Evernote app contained a basic food dairy, set up in the same way as the paper diary. This photo-based diary contained defined sections to record each eating occasion with space to write a short summary of the food and drink consumed. It also contained a designated space to add photographs of the meal before and after consumption, to estimate the proportion of food consumed. Participants were shown how to use Evernote, to photograph effectively and record details of each entry underneath each photo. As with the written diaries, they were provided with tailored instructions and examples; these focused on the electronic aspects of recording intake. Participants were asked to complete the photo-based diary on 4 non-consecutive days, including a weekend day. After all diet records were completed, all child and parent participants were invited to group interviews to gain feedback on the photo-based diary, particularly with respect to its ease of use compared to the paper diary. Child participants were invited to 1 interview and parents to a separate interview afterwards, so that any additional topics of interest that arose from the child interview could be covered in the parent interview.

### 2.3. Food Record Coding and Statistical Analyses

A trained researcher entered data from all food diaries into Kai-culator, a bespoke dietary assessment software application developed by the Department of Human Nutrition, which uses the 2014 version of the New Zealand food composition database ‘NZ FOODfiles’ (Version 1.08d, Department of Human Nutrition, University of Otago, Dunedin, New Zealand).

The photographs obtained from the photo-based diary were used to augment written information provided by participants, including pictures of additional helpings, if present. If foods in the diaries were not in the database, a similar product was substituted. For example, one participant consumed a German-made chocolate biscuit. The nutrient data for this product were searched for on Google, and the closest matched New Zealand biscuit was used.

When insufficient data were available to match food exactly, standardized substitutions were assumed. For example, if a ‘handful’ or ‘scoopful’ of hot chips was recorded in the food diary, the quantity was estimated if there was a photo, using standard portion photos developed for use in New Zealand national nutrition surveys, or it was assumed to be equal to 144 g, a typical portion size for this age group in New Zealand, using data from the most recent national survey. Nutrient information was obtained for all participants from all diaries, and simple descriptive statistics (mean and SD) were undertaken. Spearman’s correlation coefficients (SCC) were calculated to assess agreement between the nutrient information obtained from the electronic and written food diaries. As suggested by experts in the field of dietary assessment methodology [10,11], SCC of 0.3 and above were considered acceptable.

## 3. Results

All participants completed at least three days for both the paper and photo-based diaries. A total of 64 days of entries from a possible 64 was included in the final analysis of the electronic diaries and 58 days for the written diaries. The results from Table 1 show that nutrient intakes generated from the photo-based diary were similar to those from the written food diary for all participants together and for boys and girls separately. SCCs for all participants for all nutrients, with the exception of sugars, were above 0.3. SCCs conducted for boys and girls separately showed similar results with the exception of sugars, where the SCC for girls was 0.3. Intakes from the written and photo-based diaries were broadly comparable to, but lower than those intakes from children who participated in the most recent Children’s Nutrition Survey in New Zealand (CNS) in 2002 [12]. When data from boys and girls were combined, carbohydrate intake was 30 g higher from the written diary (around 5% of a child’s energy intake) compared to the photo-based diary. Boys had a higher energy intake than girls, as expected.

The information provided in the photo-based diary made data entry easier and more straightforward than the written diary for several reasons. Firstly, typed information was easier to read than the children’s handwriting. Photos in the photo-based diary provided additional information compared to what children included in some of the written diaries. Examples of this were a lack of detail in the written entries on foods that do not make up a main component of a meal, e.g., not documenting tomato sauce when consuming chips, grated cheese added to the top of pies, custard or cream added to a cake, or the exact composition of sandwiches. When reported in both diaries, some cheese sandwich photographs from the electronic food diaries showed additional food information not commonly reported in the written food diaries, such as tomatoes, vegetables or salad and bread type. Similarly, the amounts of butter added to toast or nuts contained in a handful were able to be more accurately estimated from photographs than from the paper diaries. Investigation of the photographs showed that other foods such as gravy or sauces tended not to be reported in the written diary or in the photo-based diary, even though these could be seen in the photographs. Drinks were not always reported by all participants, even when a can or carton of a drink was photographed as part of a meal.

Five children and three parents attended the group interviews, and the other child and parent participants provided written feedback on the photo-based diary. Individual feedback from all participants and their parents showed that the photo-based diary was more acceptable than the written diary, as it reduced the burden of writing everything down. If the children were unable to record everything in writing at the time of consumption, e.g., at a birthday party or a family gathering, they appreciated being able to photograph the food and add additional information when they returned home. There were several other examples of this, particularly relating to busy time periods. One was where participants made their school lunch the night before. They took a photo then, and as they did not have time to complete a full diary entry at lunchtime, they took a picture of the leftovers at the time and provided the written text that evening.

Parents and children reported that the iPod had the advantage of novelty over the written diary, which led to the children being more motivated to fill in the photo-based diary, compared to the written diary. Specific comments from the children were that the iPod is ‘a lot more portable than the paper diary’, ‘writing everything down is boring’ and ‘the autocorrect for spelling in the iPod diary also make recording everything easy’. Two children reported that the electronic diaries were quicker to complete than the paper diaries, but the other three said that the two methods took similar amounts of time. Two participants needed help from parents to fill in the paper diary as ‘It’s neater when my mum does it’, but were able to complete the photo-based diary on their own. Similarly, all participants had some help with estimating portion sizes for the paper diaries from parents. This was corroborated by comments from parents such as ‘I did quite a lot of the writing’ and ‘My daughter was more concerned with how she was spelling things and whether people would be able to read, so I did most of the writing on the paper diaries’.

Generally, although participants preferred the photo-based diary, one participant found the keyboard on the iPod too small, and ‘struggled to type’ as it is ‘really hard because you might type the wrong letter but when you have the book you can write it correctly’. When this was discussed further, all participants thought it would be easy to complete on a larger smartphone. One participant was not allowed to use the iPod at school, but they took photos of all the food they took to school and kept leftovers and packaging of food bought at school to photograph at the end of the school day. Reponses from parents indicated that they preferred the photo-based diary as less input was needed from them than for the paper diary. When parents helped with the photo-based diary, it was with the food descriptions, not photographs, e.g., how a particular food was cooked or information on some of the ingredients in composite dishes. One participant noted that their ‘mum doesn’t like technology’, but that they were willing for them to use it for ‘important’ purposes, such as school or research; the other participants agreed with this comment.

## 4. Discussion

Electronic food diaries produced similar results to written food diaries in children aged 9–12 years. Nearly all nutrient values were comparable between the food diaries. The only substantial difference was a higher carbohydrate intake from the iPod diaries. As we found acceptable correlations of 0.3 or above [10,11] for all nutrients except sugar, the results indicate that the photo-based diary shows promise as a valid dietary assessment tool for this age group. However, we must interpret these results with caution, due to the small sample size, and a further, larger validity study is needed to confirm these results. However, these results strengthen current research that suggests that technology may be an appropriate tool to measure nutrient intake [8,9].

In terms of comparing our study results with those from the only available nationally-representative dataset, the results are broadly comparable, although some small differences are seen. This is likely to be due to the different time periods in which data were collected as the CNS data are from 2002. Data from the two most recent adult nutrition surveys suggest that major changes in food consumption have occurred in New Zealand over the 10-year period between these surveys [13], which may account for some of the differences seen. The CNS used one 24-h recall from a parent to collect dietary information, which may have contributed to the observed differences.

Photo-based collection methods are becoming more feasible due the wide-spread use of smartphones by people of all ages, including older children. Current smartphones with high quality cameras are now available and inexpensive [13], meaning that this is technology accessible to most people, and smartphones, or other devices, can be provided by researchers to those without. iPods were provided for children as they do not generally have access to smart phones. The photo-based diary is suitable for and has been tested on a variety of Android-based mobile phones, as well as iPhones and iPads.

Electronic food diaries have the benefit of providing extra details not always included in written food diaries. Items such as mayonnaise on chips are often omitted from food diaries, possibly resulting in underreporting of energy intake [7,8,9]. As such foods can be seen in photos, they can be accounted for by the researcher, possibly leading to more accurate results [7,8,9], and this may explain the additional carbohydrate intakes seen in the iPod data. An important advantage of electronic food diaries is that it is that researchers can more easily gain enough information to enter food records into the database for more accurate nutrient intake estimates. When using the paper food diary, parents often had to be asked extra questions about their child’s food intake because not enough detail was provided, e.g., how many slices of bread a sandwich had or how many potatoes were eaten with dinner. The photo-based diary overcomes this because it can be seen clearly in the photos how much food was eaten. This reduces researcher and participant burden. A further advantage is that leftovers can be photographed. Children often do not eat all that is provided; recording these results accurately is important.

Another important benefit of taking photos of food and drinks is that the burden of estimating portion size is transferred from the participant to the researcher. Children find it difficult to accurately estimate the amount of food they are consuming, resulting in inaccurate nutrient intake results [9]. Researchers and dietitians who have training in portion size estimation have been shown to produce estimates that highly correlate with weighed food portions [14]. Electronic food diaries are therefore likely to have a high accuracy level.

Study limitations included the small sample size and representation of New Zealand’s population; some results and feedback may not be generalizable to all New Zealand children. During this study, furthermore, children sometimes forgot to take the iPod with them and were forced to rely on memory to write down what they had eaten at a later time. This may have reduced the accuracy of the results as no photo was provided and information was not recorded straight away. However, the same problem occurred with the written food diary, showing that the photo-based diary should be no less accurate than a written diary. An interesting observation was some parents and one school seemed to resist technology use, such as portable devices, but these devices were permitted to be used for academic or research purposes. This challenge would require further thought in a larger validation study.

A further possible limitation is that by asking all participants to complete the paper diary first, followed almost immediately (usually 4–7 days later) by the photo-based diary could lead to over-estimation of the agreement between methods. Indeed, experts in the validation of dietary methods recommend that a sufficient time period elapse between completion of the dietary assessment methods used to minimise learning effects. However, most of these recommendations come from the field of FFQ validation [11], where the learning effects are primarily related to test-retest reliability. As the principal of the two methods tested in this study was essentially the same, the information provided around writing down information in both diaries was the same and the only additional instruction for the photo-based dairy was based on the photos, learning effects should be minimal between the two methods. However, we acknowledge that (a) randomising participants as to which diary they complete first and (b) using a greater washout period may have led to lower agreement between methods. It is important to note that this study is not a formal validity study. It was designed to initially assess the feasibility of the diary as a first step before a larger, formal validity study with a larger sample size and appropriate statistical methods such as Bland–Altman.

In conclusion, this study supports the use of electronic food diaries in children, pending further formal validation. Electronic food diaries produce comparable results to written food diaries, have the advantage of being more fun for participants to fill in and provide more information to facilitate data entry for researchers. Using iPods reduces the burden on participants by replacing the need to write down comprehensive descriptions of food consumed with information from photos and may have an important role in child research in the future.

## 5. Conclusions

This novel method of dietary data collection reduces burden for participants and researchers, and allows for the more accurate coding of diet records, as it requires less estimation around portion sizes from participants [4]. The detail available from the photographs makes coding decisions more straightforward than from traditional diaries. As participants reported enjoying completing the electronic diaries, greater compliance may be seen in larger studies compared to paper diaries.

## Figures and Tables

**Table 1:** Summary of key nutrients from written and photo-based diaries (*n* = 16) compared to data from the Children’s Nutrition Survey in New Zealand (CNS) plus SCC for written and photo-based diaries.

	Written Food Diary ^1^			Photo-Based Diary ^1^			CNS Data 7–10-Year-Olds ^2^		SCC ^3^
	All Participants	Males (*n* = 8)	Females (*n* = 8)	All Participants	Males (*n* = 8)	Females (*n* = 8)	Males	Females	All Participants
Energy (kJ/d)	6825 ± 853	7502 ± 1034	6444 ± 684	6615 ± 1006	7309 ± 162	6273 ± 1079	9015	7844	0.73 **
Protein (g/d)	62.8 ± 12.0	63.0 ± 15.9	58.9 ± 8.18	66.1 ± 10.5	71.7 ± 8.82	67.1 ± 14.2	73	64	0.32
Total fat (g/d)	64.1 ± 13.6	67.4 ± 19.0	59.0 ± 7.75	63.3 ± 11.1	70.3 ± 2.47	61.9 ± 13.1	81	70	0.61 *
Carbohydrate (g/d)	203 ± 27.4	237 ± 8.35	196 ± 31.1	189 ± 40.0	210 ± 11.9	171 ± 45.5	286	251	0.59 *
Fibre (g/d)	19.0 ± 4.60	24.7 ± 3.80	17.0 ± 3.86	18.4 ± 4.53	19.0 ± 3.07	17.9 ± 5.17	19.1	16.8	0.63 *
Saturated fat (g/d)	28.3 ± 5.94	29.6 ± 7.60	25.4 ± 3.53	25.9 ± 5.90	31.0 ± 3.80	26.5 ± 6.52	35.8	30.5	0.44 *
Sugar (g/d)	87.3 ± 18.2	96.9 ± 18.4	94.8 ± 21.0	80.2 ± 18.4	80.8 ± 5.54	68.5 ± 16.0	130	115	−0.03
Calcium (mg/d)	737 ± 280	592 ± 217	704 ± 153	727 ± 222	608 ± 47.1	796 ± 195	806	653	0.83 **
Vitamin C (mg/d)	77.4 ± 32.1	107 ± 18.0	56.2 ± 15.1	71.1 ± 39.3	68.9 ± 30.8	46.1 ± 22.2	123	103	0.35

^1^ Mean ± SD, ^2^ New Zealand Children’s Nutrition Survey data 2002, *n* = 3275, mean, ^3^ SCC *=* Spearman’s correlation coefficients for written and photo-based food diaries, * *p* < 0.05, ** *p* < 0.0001.
